# Hypoxia-Induced Glioma-Derived Exosomal miRNA-199a-3p Promotes Ischemic Injury of Peritumoral Neurons by Inhibiting the mTOR Pathway

**DOI:** 10.1155/2020/5609637

**Published:** 2020-10-13

**Authors:** Jian-Lan Zhao, Bo Tan, Gong Chen, Xiao-Ming Che, Zhuo-Ying Du, Qiang Yuan, Jian Yu, Yi-Rui Sun, Xiao-Mu Li, Jin Hu, Rong Xie

**Affiliations:** ^1^Department of Neurosurgery, Huashan Hospital, Fudan University, 12 Wulumuqi Road (M), Shanghai 200040, China; ^2^Neurosurgical Institute of Fudan University, 12 Wulumuqi Road (M), Shanghai 200040, China; ^3^Department of Endocrinology, Zhongshan Hospital, Fudan University, 180 Fenglin Road, Shanghai 200032, China

## Abstract

The underlying molecular mechanisms that the hypoxic microenvironment could aggravate neuronal injury are still not clear. In this study, we hypothesized that the exosomes, exosomal miRNAs, and the mTOR signaling pathway might be involved in hypoxic peritumoral neuronal injury in glioma. Multimodal radiological images, HE, and HIF-1*α* staining of high-grade glioma (HGG) samples revealed that the peritumoral hypoxic area overlapped with the cytotoxic edema region and directly contacted with normal neurons. In either direct or indirect coculture system, hypoxia could promote normal mouse hippocampal neuronal cell (HT22) injury, and the growth of HT22 cells was suppressed by C6 glioma cells under hypoxic condition. For administrating hypoxia-induced glioma-derived exosomes (HIGDE) that could aggravate oxygen-glucose deprivation (OGD)/reperfusion neuronal injury, we identified that exosomes may be the communication medium between glioma cells and peritumoral neurons, and we furtherly found that exosomal miR-199a-3p mediated the OGD/reperfusion neuronal injury process by suppressing the mTOR signaling pathway. Moreover, the upregulation of miRNA-199a-3p in exosomes from glioma cells was induced by hypoxia-related HIF-1*α* activation. To sum up, hypoxia-induced glioma-derived exosomal miRNA-199a-3p can be upregulated by the activation of HIF-1*α* and is able to increase the ischemic injury of peritumoral neurons by inhibiting the mTOR pathway.

## 1. Introduction

Glioblastoma multiforme (GBM) is the most common primary brain malignancy in adults with consistently very poor outcomes [1]. Peritumoral hypoxia of solid tumors develops due to rapid tumor growth exceeding the vascular supply capabilities and/or tumor vasculature malfunctioning [2]. Hypoxia is one of the most significant characteristics of GBM [3], and pseudopalisading necrosis with surrounding hypoxia is identified as a hallmark pathognomonic feature [4]. It has been reported that the peritumoral hypoxic microenvironment could promote the growth of glioma cells [5]; however, its underlying molecular mechanisms are still not clear.

Exosomes are 40–100 nm nanosized vesicles released into the extracellular space from many cell types, including blood cells [6], and have been reported to play important roles in tumor progression [7]. However, the mechanism of exosomes in the occurrence and/or regulation of hypoxia-induced glioma cell proliferation and neuronal injury remains unclear. MicroRNAs (miRNAs), small noncoding RNAs of 17~24 nucleotides, are able to regulate diverse biological processes through downregulation (sometimes upregulation) of the expression of target proteins [8]. miRNAs have been confirmed to be altered by hypoxia to target crucial oncogenes and tumor suppressors, which are the hallmarks of tumorigenic processes [9]. Valadi et al. reported the intercellular transfer of miRNAs via exosomes [10]. Thus, it is reasonable to hypothesize that exosomal miRNAs participate in hypoxic exosomal phenomena. However, such phenomena have rarely been studied.

The mammalian target of rapamycin (mTOR) signaling pathway has been confirmed to play a central role in cell metabolism, growth, differentiation, development, and survival [11]. The mTOR pathway was constructed by various components, including its upstream molecular Akt and its downstream factor S6K [12]. The specific function of mTOR is regulated by different signal pathways [13]. By applying an oxygen-glucose deprivation (OGD) model to mimic ischemic-reperfusion injury in vitro and using dMCAO rat models in vivo, we found that mTOR is a crucial signaling pathway in the protection against cerebral ischemia-reperfusion injury [14–16]. Thus, we hypothesized that the mTOR signaling pathway might be involved in hypoxic peritumoral neuronal injury in glioma and then induce the proliferation of glioma cells.

## 2. Materials and Methods

### 2.1. Glioma Samples and Image Data

Glioma samples were obtained from patients who were pathologically diagnosed with primary glioma at Huashan Hospital, Fudan University in 2017.1-2017.12. Image data, including MRI, were also retrospectively collected. This study strictly followed the Declaration of Helsinki and was approved by the Ethical Review Boards of Fudan University Huashan Hospital. Informed consent was obtained from all individual participants.

### 2.2. Hematoxylin-Eosin (HE) and Hypoxia-Induced Factor-1*α* (HIF-1*α*) Staining

Protocols for HE [17] and HIF-1*α* [18] staining have been described previously and are briefly described in Supplementary Methods 1.

### 2.3. Cell Line and Cell Culture

HT22 normal mouse hippocampal neuronal cells were obtained from David Schubert (Salk Institute, San Diego, CA). C6 glioma cells were obtained from the American Type Tissue Collection (Rockville, MD). HT22 and C6 glioma cells were normally cultured and are briefly described in Supplementary Methods 2. In our study, the HT22 and C6 cell lines were cocultured in either direct or indirect systems. Protocols for these coculture systems were previously described [19].

### 2.4. Primary Neuronal Cultures

This study strictly followed the ARRIVE guideline and was approved by the Ethical Review Boards of Fudan University Huashan Hospital. Primary neuronal cultures were prepared using timed-pregnant Sprague-Dawley rats (E18, Charles River Laboratories International, Wilmington, MA) as previously reported [20]. This information is briefly described in Supplementary Methods 3.

### 2.5. Oxygen-Glucose Deprivation (OGD)/Reperfusion

The protocols have been described previously [15] and are briefly described in Supplementary Methods 4.

### 2.6. 3-[4,5-Dimethylthiazol-2-yl]-2,5-diphenyltetrazolium Bromide (MTT) Assay

The MTT assay is based on the protocol described previously [21] and is briefly described in Supplementary Methods 5.

### 2.7. Lactate Dehydrogenase (LDH) Release Assay

Cell viability was detected by LDH release using an LDH cytotoxicity assay kit (Cayman Chemical, Ann Arbor, MI) according to the manufacturer's protocol. LDH activity was quantified by measuring absorbance at 490 nm with a microplate reader (Hangzhou, China). The ratio of released LDH to total LDH was calculated and presented as a relative LDH release compared to LDH release in nontreated cells.

### 2.8. Exosome Isolation and Identification

Exosomes were isolated from a postconditioned medium by successive centrifugation after 24 hours of culture in the presence of vesicle-depleted FBS (Wisent Inc.), as previously described [22]. To determine whether exosomes were successfully isolated, transmission electron microscopy (TEM) (NanoSight Ltd., Amesbury, UK) and detection of the expression of CD81 by Western blotting (see below) were performed. For TEM, the protocol is briefly described in Supplementary Methods 6. Sizing of exosomes was performed by nanoparticle tracking analysis using the NanoSight LM10 instrument (NanoSight Ltd., Amesbury, UK) and ZetaView (Particle Metrix, Meerbusch, Germany).

### 2.9. Microarray Analysis

Total RNA was extracted using TRIzol reagent (Invitrogen, Carlsbad, CA, USA), and according to the manufacturer's protocols, we conducted microarray analysis (Rat miRNA Microarray, Release 21.0, Aksomics, Shanghai, China) to detect the altered expression of miRNAs.

### 2.10. Lentiviral Vector Generation, Titration, and Gene Delivery

We used a lentiviral vector containing miR-199a-3p shRNA to inhibit miR-199a-3p expression (control: scramble shRNA). Moreover, we commercially constructed lentiviral vectors expressing S6K and HIF-1*α*. We used a 3-plasmid system for lentivirus packaging as detailed in our previous study [15]. The virus particles were tittered with the TCID50 method as described previously [23]. The protocols are briefly described in Supplementary Methods 7. The manufacturers and catalog numbers of the lentiviral vectors and lentiviral plasmid backbones used are listed in Supplementary Table S1.

### 2.11. Other Transfection Reagents

miR-199a-3p mimics and negative controls of miR-199a-3p mimic were purchased from Thermo Fisher Scientific Inc. (miR-199a-3p mimics: MC11779, negative control: 4464058). The miR-199a-3p mimics and negative controls (1 *μ*g/ml) were directly added to the cell cultures at the indicated concentration 24 hours before OGD induction.

### 2.12. Protein Preparation and Western Blot Analysis

The protein levels of CD81 were examined by Western blotting. The harvested cells were homogenized in cold cell extraction buffer (Cat# FNN0001, Invitrogen, Carlsbad, CA, USA) containing 1 mM PMSF and protease inhibitor cocktail (1 : 20, Cat# P-2714, Sigma). The homogenates were centrifuged at 4°C for 20 min at 13000 rpm. The supernatant was used for Western blotting and is briefly described in Supplementary Methods 8. The manufacturers and catalog numbers of all primary antibodies used are listed in Supplementary Table S2.

### 2.13. Reverse Transcription-Quantitative Polymerase Chain Reaction (RT-qPCR)

The RT-qPCR protocol is briefly described in Supplementary Methods 9, and the primers used in this research are listed in Supplementary Table S3.

### 2.14. Statistical Analysis

All results obtained from at least 3 independent experiments were performed, *n* = 14‐21/group. GraphPad Prism 6.0 software was used for statistical analyses. One-way or 2-way ANOVA was used followed by the Fisher least significant difference post hoc test. Tests were considered significant at *P* values < 0.05. Data are presented as the mean ± standard errors (SE).

## 3. Results

### 3.1. Evidence of Hypoxia Was Observed in Both Glioma and Its Surrounding Parenchyma

**Figure 1:**
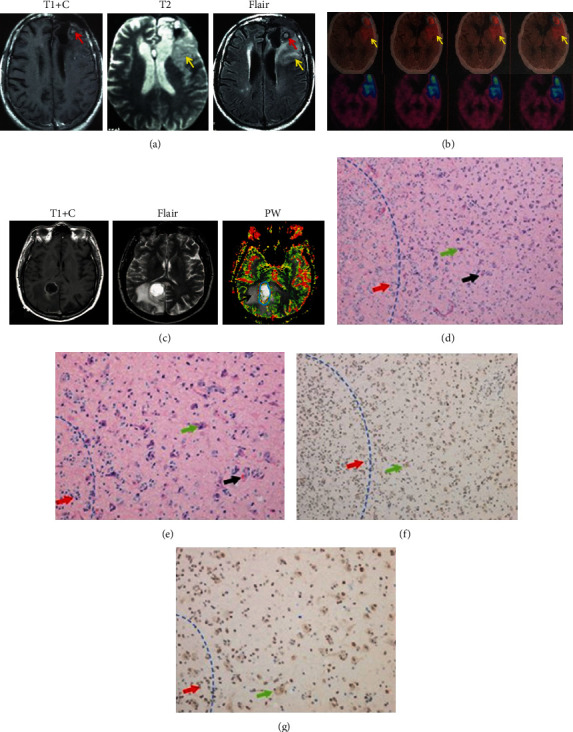
The peritumoral hypoxic area overlapped with the cytotoxic edema region. (a) An anaplasia astrocyte glioma (WHO III) sample showed a hyperintense signal in T1 contrast scanning (red arrow), and peritumoral edema, which indicated potential hypoxia, was detected in T2 and Flair scanning images (yellow arrow). (b) When the patient received 18-FDG PET/CT scanning, hypoxia was found in the tumor region (blue ring) and peritumor edema region (yellow arrow). Moreover, when using 18F-FMISO, hypoxia (blue area) shared the same region as T2 and Flair scanning, which further indicated that peritumoral edema was closely correlated with the occurrence of hypoxia around the tumor. (c) In another anaplasia astrocyte glioma (WHO III) patient, T1+contrast, Flair, and MR PW studies revealed that regions of edema featured low perfusion characteristics (c). The analyzed sample was obtained from the region between the tumor and normal parenchyma. The blue dotted line shows the border between the tumor and the normal brain tissue. (d, e) HE staining (×100 and ×400) of the sample. Glioma cells (red arrow) invaded normal neuronal cells (green arrow). Neurons with morphological impairments can also be detected. The black arrow indicates the microthrombus, which was considered a characteristic of hypoxia. (f, g) HIF-1*α* staining (×100 and ×400) of the sample. Positive HIF-1*α* staining was identified in tumor cells (red arrow) and peritumor normal neurons (green arrow), which indicated that hypoxia occurred in the peritumoral region. However, HIF-1*α* staining was negative in neurons located away from the tumor.

Multimodal radiological assessments were performed for patients with glioma to verify whether hypoxia occurred in glioma and its surrounding parenchyma as previously reported. In MRI, the tumor lesion is best defined on contrast-enhanced T1W sequence, while paratumoral vasogenic and/or cytotoxic edema can be clearly identified as hyperintense signals on T2 and Flair sequences (Figure 1(a)). Further ^18^F-FDG and ^18^F-FMISO PET/CT scannings were performed in this glioma patient and clearly showed that the regions of paratumoral hypoxia were the same as the regions of edema identified on T2 and Flair MRI images, which demonstrated a close relationship between paratumoral edema and the occurrence of hypoxia (Figure 1(b)). Regions of edema showed low perfusion features on MR PW studies (Figure 1(c)).

Tumor samples from surgical resection were processed by HE and HIF-1*α* staining (Figures 1(d) and 1(e)). Normal neurons mixed with high-grade glioma (HGG) cells within the paratumoral area were observed by HE staining, indicating the invasive growth of glioma cells. In addition, microthrombi were also observed. The density of positive HIF-1*α* staining was higher in tumoral areas than in normal parenchyma, which confirmed the existence of hypoxia in glioma and its surrounding parenchyma (Figures 1(f) and 1(g)) and also suggested that glioma cells were growing within and around hypoxic environments.

### 3.2. Hypoxia Promoted C6 Cell Proliferation and HT22 Neuron Injury via Exosomes and Exosomal miR-199a-3p

**Figure 2:**
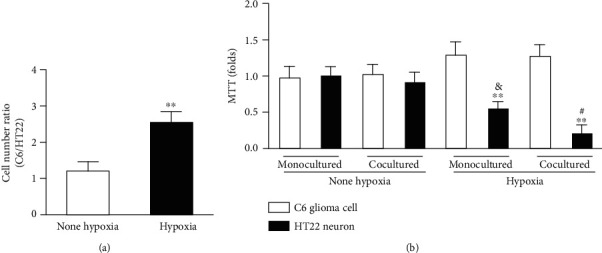
Effects of hypoxia on the proliferation of C6 and HT22 cells in the coculture system. (a) In the direct coculture system, the ratio of C6/HT22 cells was significantly higher under hypoxia after 3 days. ^∗∗^*P* < 0.01 vs. no hypoxia. (b) In the indirect coculture system, the proliferation ability of HT22 and C6 cells was similar under normal conditions, but when hypoxic conditions were applied, the MTT level of HT22 cells was lower than that of C6 cells in both the mono- and coculture systems. Moreover, HT22 cells had much lower MTT levels in the coculture system than in the monoculture system. ^∗∗^*P* < 0.01 vs. C6 glioma cells; ^#^*P* < 0.05 vs. HT22 cells monocultured under hypoxic conditions. ^&^*P* < 0.05 vs. HT22 cells monocultured under none hypoxic conditions. *n* = 14‐21/group in at least 3 independent experiments.

In a direct coculture system, the ratio of C6/HT22 cells was significantly increased after hypoxia treatment (Figure 2(a)). C6 and HT22 cells were further cultured separately in both monoculture and indirect coculture systems. The proliferation ability of HT22 cells was decreased after hypoxia treatment in the monoculture system but was significantly further suppressed in the indirect coculture system with C6 cells. In addition, the MTT of C6 cells in hypoxia condition were higher than those in none hypoxia environment (Figure 2(b)). However, the question remained of what caused the phenotype that in either the direct or indirect coculture system, the growth of HT22 cells was suppressed by C6 cells under hypoxic conditions. In the study, we identified exosomes and exosomal miRNA as the potential underlying mechanism.

**Figure 3:**
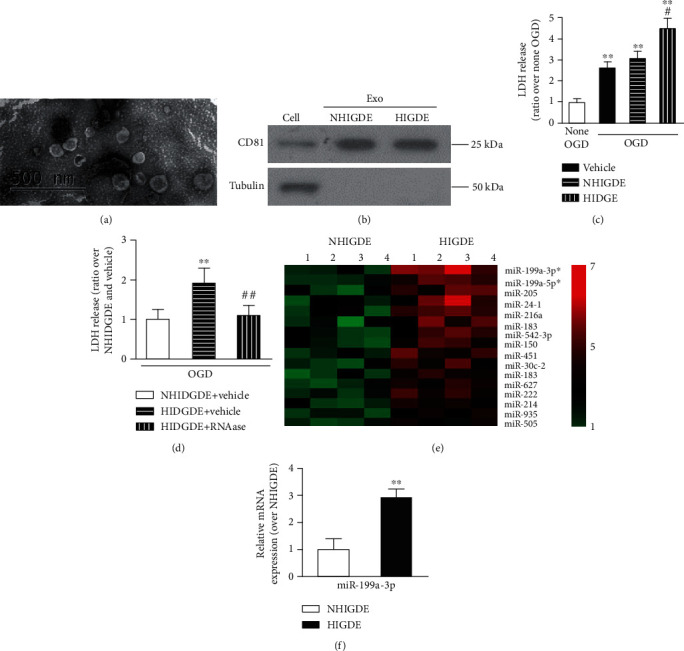
HIGDE aggravated OGD injury in normal HT22 cells, and miRNA-199a-3p was identified as a target miRNA that induced the different hypoxic injury effects between HIGDE and NHIGDE. (a) Exosomes were isolated from C6 glioma cells and were identified by TEM. Isolated exosomes were consistent in size and shape. (b) The expression of CD81 was clearly detected in isolated HIGDE/NHIGDE and C6 cells. (c) After applying OGD/reperfusion injury, the LDH level in HT22 cells was significantly increased with HIGDE treatment in comparison to NHIGDE and vehicle. ^∗∗^*P* < 0.01 vs. no OGD; ^#^*P* < 0.05 vs. NHIGDE and vehicle. (d) After applying OGD/reperfusion injury, the LDH level of HT22 cells treated with HIGDE+RNase was lower than that in cells treated with HIGDE+vehicle but was similar as to that in cells treated with NHIGDE+vehicle. ^∗∗^*P* < 0.01 vs. NHIGDE+vehicle; ^##^*P* < 0.01 vs. HIGDE+vehicle. (e) MicroRNA microarray analysis indicated that among the sixteen miRNAs that were expressed significantly higher in HIGDE than NHIGDE, the most upregulated one was miRNA-199a-3p (*P* < 0.01, fold change > 2). (f) Confirmed by RT-qPCR, the expression of miRNA-199a-3p was almost two times higher in HIGDE than NHIGDE. ^∗∗^*P* < 0.01 vs. NHIGDE. *n* = 14‐21/group in at least 3 independent experiments.

First, exosomes were isolated from the medium of C6 cells cultured with or without hypoxia (1% oxygen) treatment (hypoxia-induced glioma-derived exosomes (HIGDE) and non-hypoxia-induced glioma-derived exosomes (NHIGDE)). Their size and morphology were identified by TEM (Figure 3(a)). The presence of CD81 (a specific marker of exosomes) in isolated exosomes was confirmed using Western blotting (Figure 3(b)). LDH release after OGD/reperfusion injury in HT22 cells was significantly increased with HIGDE treatment but remained the same after NHIGDE treatment (Figure 3(c)). These results suggest that HIGDE might aggravate hypoxia-induced neuronal injury.

Then, RNase was used to degrade RNA components in exosomes to further investigate the potential mechanism of HIGDE-induced OGD/reperfusion injury in HT22 cells. The level of LDH release in HT22 cells with HIGDE treatment was significantly lower after RNase treatment compared with that in the non-RNase-treated (vehicle) group and was similar to that in the NHIGDE group (Figure 3(d)). The microRNA microarray analysis revealed sixteen highly expressed miRNAs in HIGDE than NHIGDE (Figure 3(e)). Among them, miRNA-199a-3p was the most highly expressed, with approximately 3 times the 1level in HIGDE. This was also confirmed by RT-qPCR (Figure 3(f)).

### 3.3. Exosomal miR-199a-3p Mediated HIGDE-Induced OGD/Reperfusion Injury in Primary Neurons

**Figure 4:**
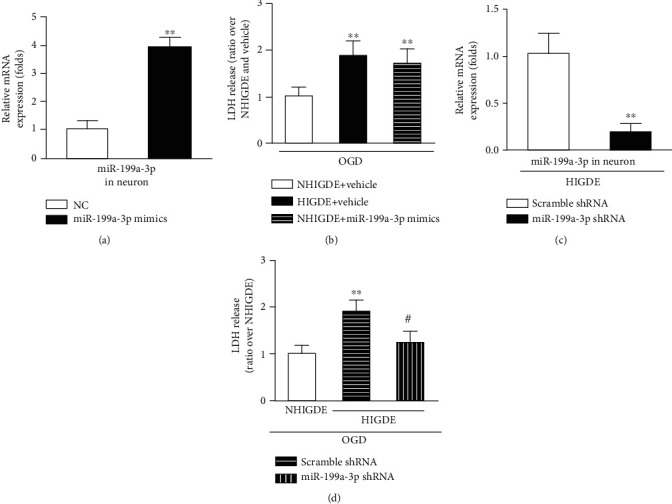
miRNA-199a-3p could aggravate OGD injury in primary cultured neurons. (a) RT-qPCR confirmed that the constructed miRNA-199a-3p mimics were successfully transformed into primary cultured neurons. ^∗∗^*P* < 0.01 vs. NC (premiRNA negative control). (b) After OGD/reperfusion injury, the LDH level of primary cultured neurons treated with NHIGDE+miRNA-199a-3p mimics was higher than that in cells treated with NHIGDE+vehicle but was similar to that in cells treated with HIGDE+vehicle. ^∗∗^*P* < 0.01 vs. NHIGDE+vehicle. (c) RT-qPCR validated that the expression of miRNA-199a-3p in HIGDE from C6 cells was knocked down by miRNA-199a-3p shRNA. ^∗∗^*P* < 0.01 vs. scramble shRNA. (d) After OGD/reperfusion injury, the LDH release level by primary cultured neurons treated with HIGDE+miRNA-199a-3p shRNA or NHIGDE was lower than that of primary cultured neurons treated with HIGDE+scramble shRNA. ^∗∗^*P* < 0.01 vs. NHIGDE; ^#^*P* < 0.05 vs. HIGDE+scramble shRNA. *n* = 11‐16/group in at least 3 independent experiments.

To elucidate the effects of miRNA-199a-3p on ischemic-reperfusion injury of neurons, we transfected primary cultured neurons with miRNA-199a-3p mimic or control miRNA (Figure 4(a)). The LDH assay showed that primary cultured neurons with NHIGDE+miRNA-199a-3p mimics suffered more severe OGD/reperfusion injury, which was similar to the effects of HIGDE (Figure 4(b)). In addition, miRNA-199a-3p shRNA was used to knock down the expression of miRNA-199a-3p in neuronal cells (Figure 4(c)). LDH release levels suggested that the ischemia injury caused by HIGDE was blocked by miRNA-199a-3p shRNA (Figure 4(d)).

### 3.4. miRNA-199a-3p Aggravated Neuronal Injury with HIGDE Administration by Suppressing the mTOR Signal

**Figure 5:**
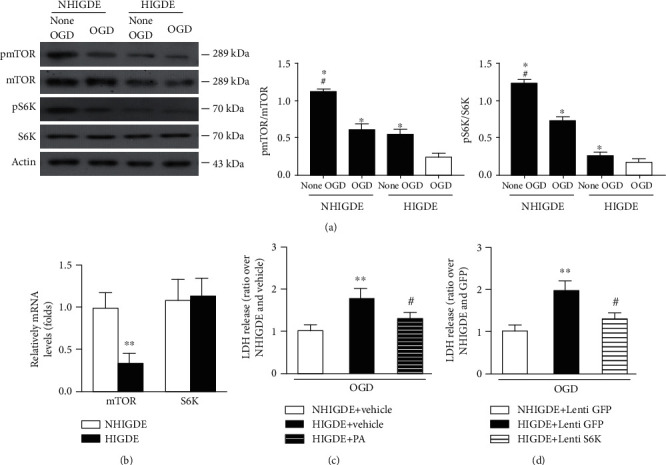
miRNA-199a-3p in HIGDE aggravated neuronal injury by suppressing the mTOR signaling pathway. (a) Representative protein bands and quantification of pmTOR, mTOR, pS6K, and S6K from primary cultured neurons treated with NHIGDE or HIGDE after or in the absence of OGD. ^∗^*P* < 0.01 vs. HIGDE+OGD; ^#^*P* < 0.05 vs. NHIGDE+OGD. (b) The mRNA level of mTOR in primary cultured neurons was lower when the cells were treated with HIGDE than with NHIGDE; however, the expression of S6K was similar. ^∗∗^*P* < 0.01 vs. NHIGDE. (c) After OGD/reperfusion injury, when the cells were treated with PA, the LDH level in primary cultured neurons treated with HIGDE+PA (an mTOR activator) distinctly decreased compared with that in the neurons treated with HIGDE+vehicle, similar to the neurons treated with NHIGDE+vehicle. ^∗∗^*P* < 0.01 vs. NHIGDE+vehicle; ^#^*P* < 0.05 vs. HIGDE+vehicle. (d) The LDH level of primary cultured neurons treated with HIGDE+Lenti S6K (a downstream effector of mTOR) after OGD also decreased compared with that in neurons treated with HIGDE+Lenti GFP, and was similar to that in neurons treated with NHIGDE+Lenti GFP. ^∗∗^*P* < 0.01 vs. NHIGDE+Lenti GFP; ^#^*P* < 0.05 vs. HIGDE+Lenti GFP. *n* = 14‐21/group in at least 3 independent experiments.

NHIGDE and HIGDE from C6 cells were isolated and administered to the medium of primary cultured neurons. After the OGD/reperfusion treatment, the expression of pmTOR, mTOR, and pS6K was clearly decreased in comparison to that in non-OGD neurons (Figure 5(a)). The mRNA level of total mTOR in primary cultured neurons was also significantly lower with HIGDE administration than with NHIGDE administration, but the mRNA level of total S6K remained stable (Figure 5(b)). After OGD/reperfusion injury, LDH released from the neurons treated with phosphatidic acid (PA) plus HIGDE, similar to the neurons treated with NHIGDE+vehicle, was lower than that in the neurons treated only with HIGDE+vehicle (Figure 5(c)). Moreover, when S6K was overexpressed, the OGD/reperfusion injury of primary neurons treated with HIGDE was significantly decreased in comparison to that in neurons treated with HIGDE without S6K overexpression (Figure 5(d)).

**Figure 6:**
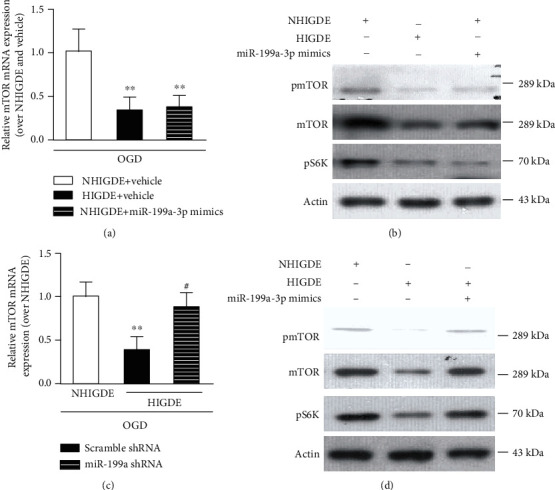
NHIGDE could aggravate OGD injury in primary neurons by overexpressing miRNA-199a-3p. (a, b) When primary neurons were treated with NHIGDE+miRNA-199a-3p mimics, the mRNA and protein levels of the mTOR pathway in primary neurons decreased compared with those in neurons treated with NHIGDE+vehicle, similar to neurons treated with HIGDE+vehicle after applied OGD/reperfusion. ^∗∗^*P* < 0.01 vs. NHIGDE+vehicle. In contrast, (c) before and (d) after OGD/reperfusion injury, the mRNA and protein levels of the mTOR pathway were higher in primary neurons cultured with HIGDE from C6 cells treated with miRNA-199a-3p shRNA than in neurons treated with HIGDE+scramble shRNA, similar to neurons treated with NHIGDE. ^∗∗^*P* < 0.01 vs. NHIGDE; ^#^*P* < 0.05 vs. scramble shRNA+HIGDE. *n* = 14‐21/group in at least 3 independent experiments.

Next, we transfected isolated NHIGDE with miRNA-199a-3p mimics. mTOR mRNA expression after OGD/reperfusion injury was significantly decreased in primary neurons treated with NHIGDE plus miRNA-199a-3p mimics, as well as HIGDE (Figure 6(a)). In addition, mTOR, pmTOR, and pS6K expression in primary neurons treated with NHIGDE plus miRNA-199a-3p mimics was obviously decreased, as indicated by Western blotting (Figure 6(b), Supplementary Figure IA). Moreover, after OGD/reperfusion injury, the mRNA level of mTOR did not decrease in primary neurons cultured with HIGDE from these C6 cells treated with miRNA-199a-3p shRNA compared with neurons cultured with NHIGDE but was much higher than that in neurons cultured with HIGDE treated only with scramble shRNA (Figure 6(c)). Finally, when primary neurons were cultured with NHIGDE or HIGDE plus miRNA-199a-3p shRNA, the expression of pmTOR, mTOR, and pS6K was clearly higher than that in neurons treated with HIGDE plus scramble shRNA (Figure 6(d), Supplementary Figure IB).

### 3.5. The Upregulation of miRNA-199a-3p in Exosomes from C6 Glioma Cells Was Induced by Hypoxia-Related HIF-1*α* Activation

**Figure 7:**
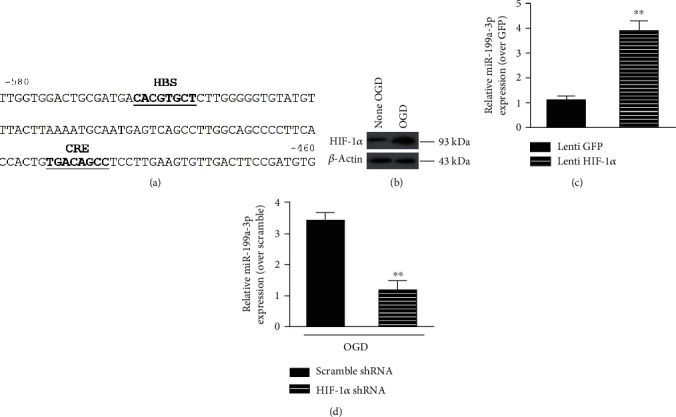
The upregulation of miRNA-199a-3p in HIGDE was mediated by hypoxia-induced HIF-1*α* activation. (a) Bioinformatics analysis identified HIF-1*α* reacting element (HRE) sequences in the upstream area of miRNA-199a1. (b) Western blotting further confirmed the upregulated expression of HIF-1*α* in C6 cells cultured in hypoxia-treated environments. (c) The expression of miRNA-199a-3p RNA in NHIGDE+Lenti-HIF-1*α* was remarkably higher than that in NHIGDE without HIF-1*α* overexpression (Lenti GFP). ^∗∗^*P* < 0.01 vs. Lenti GFP. (d) When the expression of HIF-1*α* in C6 cells was knocked down (HIF-1*α* shRNA), the expression of miRNA-199a-3p in HIGDE decreased significantly in comparison to that in HIGDE without HIF-1*α* suppression. ^∗∗^*P* < 0.05 vs. HIGDE+scramble shRNA. *n* = 14‐21/group in at least 3 independent experiments.

HIF-1*α* is a crucial regulatory factor in the oxygen balance of mammalian cells. Our bioinformatics analysis identified HIF-1*α* reacting element (HRE) sequences in the upstream area of miRNA-199a1 (Figure 7(a)). Western blotting further confirmed the upregulated expression of HIF-1*α* in C6 cells cultured in hypoxic environments (Figure 7(b)). These findings suggested that HIF-1*α* might be activated by hypoxia and was related to miRNA-199a.

When HIF-1*α* was overexpressed in C6 cells, the expression of miRNA-199a-3p RNA in NHIGDE was remarkably higher than that in NHIGDE without HIF-1*α* overexpression (Lenti GFP) (Figure 7(c)). Conversely, when the expression of HIF-1*α* in C6 cells was knocked down (HIF-1*α* shRNA), the expression of miRNA-199a-3p in HIGDE decreased significantly in comparison to that in HIGDE without HIF-1*α* suppression (Figure 7(d)).

## 4. Discussion

In this study, we found that exosomal miRNA-199a-3p could be upregulated in a hypoxic microenvironment; via suppressing the mTOR signaling pathway, HIGDE were able to exacerbate the ischemic injury of peritumoral neurons. Although the role of miR-199a-3p in glioma progression was previously investigated, the ischemic injury mechanism of peritumoral neurons, involving miR-199a-3p, exosomes, and the mTOR signaling pathway, has rarely been studied. For in ischemic stroke, infarcted brain parenchyma also characterized by hypoxia as glioma, it was reasonable that exosomes released from ischemia brain could have the same effects as HIGDE and thus can aggravate hypoxia injury in neurons around the infarcted brain. This is a novel ischemic neuronal injury mechanism.

### 4.1. Peritumor Hypoxia Is the Microenvironment That May Aggravate Ischemic Injury in Peritumoral Neurons and Promote the Invasive Growth of Tumor Cells

Hypoxia frequently occurs in solid tumors due to erratic tumor neovascularization resulting from rapid cell proliferation, which leads to poor oxygen diffusion [24]. Consistent with previous studies [25], our multimodal examinations found that areas of peritumoral edema were the same as areas of hypoxia. Because high HIF-1*α* was regarded as a biomarker of the existence of hypoxia [26], hypoxia was detected in the parenchyma surrounding glioma lesions in our study. In addition to evidence of the existence of hypoxia, the results of HE-stained surgical samples also suggested the invasive growth of glioma cells. We confirmed the existence of hypoxia in the peritumoral region. Moreover, these findings also indicated that glioma cells grew within or around hypoxic environments. Thus, we mainly focused on peritumoral neuron injury understimulated hypoxic conditions in this study.

It is widely accepted that oxygen deficiency is a major factor associated with neuronal damage and promotion of invasive growth of glioma cells [27]. In this study, we found that the proliferation ability of glioma cells was significantly higher than that of normal neuronal cells under hypoxic conditions in either a direct or indirect coculture system. These results further confirmed that the hypoxic microenvironment might be a potential factor that promotes glioma cell proliferation and, especially, exacerbates the injury of peritumoral normal neurons. But concerning that neurons do not proliferate in vivo, the ratio between C6 and HT22 may be due to a reduced number of neurons rather than an increased proliferation of glioma cell. We admitted this may be a potential explanation to the phenotype, but as shown in Figure 2(b), the MTT of C6 was statistically higher in hypoxia condition than none hypoxia environment either in mono- or cocultured system, which suggested that the ratio also may be affected by increasing C6 glioma cell proliferation ability in hypoxia. However, in this study, exosomes and exosomal miRNA were assumed as the potential underlying mechanism.

To clarify this underlying mechanism, we aimed to explore the interactions between hypoxia injury and exosomes/exosomal miRNA. Our in vitro results showed that hypoxia neuronal injury could be aggravated by the administration of HIGDE, and RNAs in exosomes were closely related to ischemic injury in normal neurons. Such exosomes (HIGDE) were different from the exosomes secreted by tumor cells under normal conditions (NHIGDE), and such differences were induced by miRNA-199a-3p. Moreover, we also verified that the upregulation of miRNA-199a-3p exacerbated hypoxic injury. On the other hand, hypoxic injury could be obviously relieved once miRNA-199a-3p was silenced. In addition, our study verified a positive relationship between the level of HIF-1*α* and miRNA-199a-3p. We concluded that HIF-1*α* could be activated by hypoxia and thus could upregulate the expression of miRNA-199a-3p, then induce ischemic injury in peritumoral normal neurons.

### 4.2. Exosomes May Be a Communication Medium between Glioma Cells and Peritumoral Neurons

Exosomes are a type of extracellular vesicles that are able to facilitate intercellular communication and crosstalk within the tumor environment. Exosomes secreted by tumor cells are now recognized to be involved in a variety of processes underlying tumor progression, including oncogenic genes and miRNAs [28]. It was confirmed that exosomes can pass the blood-brain barrier and deliver RNAs within brain tissue [29].

Our results also showed that after applying OGD to glioma cells, HIGDE, containing upregulated miRNA-199a-3p, were able to exacerbate the injury of normal neurons. In contrast, when oxygen was sufficient in C6 cells, the hypoxic injury of neuron cells treated with NHIGDE was significantly less severe, and in addition, the level of miRNA-199a-3p in NHIGDE was also lower. These findings suggested that HIGDE could be released from glioma cells into the extracellular space and enter peritumoral normal neurons under a hypoxic environment.

It was indicated that exosomes may be the connecting medium between glioma cells and normal neurons and are the pivotal factor that induces the peritumoral hypoxia neuronal injury.

### 4.3. A Novel Ischemic Injury Mechanism: miRNA-199a-3p, Exosomes, and the mTOR Signaling Pathway

As mentioned above, the upregulation of miRNA-199a-3p in exosomes of glioma cells induced by peritumoral hypoxia and HIF-1*α* activation might be the core molecular mechanism underlying the aggravated hypoxic injury of peritumoral neurons. However, the specific pathway is not clear.

Our study focused on the role of the mTOR pathway due to its well-known protective ability against damage to neurons and other tissues [15, 30]. The specific role of the mTOR pathway in neuronal cells is still under debate. Some studies have also found that mTOR activation might even be harmful after neuronal injury [31, 32]. However, our previous studies showed that the mTOR pathway played protective roles in ischemic rats [16, 33]. The relationship or interplay between mTOR and miRNA-199a-3p has been studied in cholangiocarcinoma [34], gastric cancer [35], and nonneoplastic C2C12 muscle cells [36]. Shen et al. found that miR-199a-3p in glioma cell lines can be the tumor suppressor gene on cellular proliferation via the AKT/mTOR signaling pathway [37]. Moreover, in spinal cord injury rats, activity-dependent plasticity can be modulated by miRNA-199a-3p via the PTEN/mTOR pathway [38]. It is suggested that the activities of the mTOR pathway may be regulated by miRNA-199a-3p after stroke-related ischemic injury, but to the best of our knowledge, the molecular mechanism involving mTOR and miRNA-199a-3p has rarely been investigated in the hypoxic injury of neurons.

In the current study, mTOR showed protective effects against the injury of neurons subjected to a hypoxic environment. In other words, activating the mTOR pathway might relieve the hypoxic injury of peritumoral normal neuronal cells induced by glioma cells and might inhibit the invasive growth of glioma cells in a hypoxic microenvironment.

**Figure 8:**
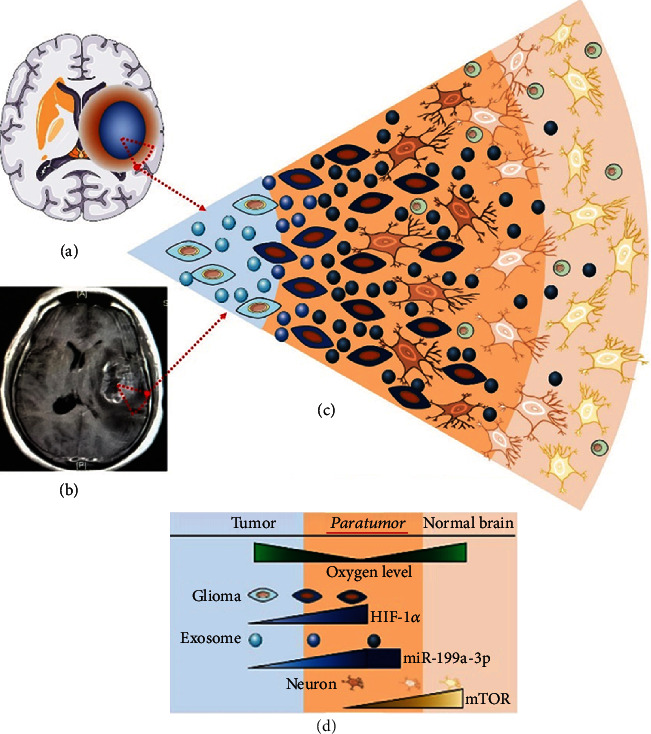
A schematic summarization of the key findings. HIGDE miRNA-199a-3p can be upregulated by hypoxia-induced HIF-1*α* activation and then is able to increase ischemic injury of peritumoral neurons by inhibiting the mTOR pathway. The region we focused on in the current study was the red triangle in (a) and (b). (a) Blue region: glioma lesion; yellow region around the lesion: hypoxia region. (b) The glioma lesion and its surrounding hypoxic region in MRI T1 enhanced scanning. (c, d) The microenvironment of the peritumoral region. Blue background: intratumoral hypoxia region. Dark yellow background: peritumoral hypoxia region. Light yellow background: normal brain nonhypoxic region. As the hypoxic condition worsened, HIF-1*α* was activated and increased the level of miRNA-199a-3p in the exosomes secreted by glioma cells (light blue ball: NHIGDE; dark blue ball: HIGDE). Then, exosomes were released and inhibited the mTOR level in peritumoral normal neurons. Thus, hypoxia-induced injuries of peritumoral neurons were aggravated, and glioma growth was also facilitated.

We used PA and overexpression of S6K to verify this mechanism. As indicated by the LDH levels, activating the mTOR signaling pathway alleviated the injury of neuronal cells under hypoxic conditions similar to peritumoral conditions in vivo, which suggested that the mechanism of the protective effects of mTOR activation against the hypoxic injury of neuronal cells was induced by HIGDE. Then, we interfered with the expression of miRNA-199a-3p. These findings suggested that miRNA-199a-3p was able to suppress the mTOR signaling pathway and thus aggravate the hypoxic injury of normal neurons induced by HIGDE (Figure 8).

There are still several limitations in this study. First, an in vivo or animal study is required to confirm our in vitro results. Moreover, a further study is required to elucidate whether the effect of miRNA-199a-3p in suppressing mTOR signaling is direct or mediated indirectly by additional factors. Finally, the molecular mechanism should be verified in other models.

## 5. Conclusion

As hypoxia worsens, hypoxia-induced glioma-derived exosomal miRNA-199a-3p can be upregulated by the activation of HIF-1*α* and is able to increase the ischemic injury of neurons by inhibiting the mTOR pathway.

## Data Availability

The data used to support the findings of this study are included within the article and the supplementary information file.

## Abbreviations

GBM: Glioblastoma multiforme
HE: Hematoxylin-eosin
HER: HIF-1*α* reacting element
HIF-1*α*: Hypoxia-inducible factor-1*α*
HIGDE: Hypoxia-induced glioma-derived exosomes
ICH: Intracerebral hemorrhage
LDH: Lactate dehydrogenase
miRNAs: MicroRNAs
mTOR: Mammalian target of rapamycin
MRI: Magnetic resonance imaging
MTT: 3-[4,5-Dimethylthiazol-2-yl]-2,5-bromide
NHIGDE: None hypoxia-induced glioma-derived exosomes
OGD: Oxygen glucose deprivation
PA: Phosphatidic acid
PW: Perfusion weighted
TEM: Transmission electron microscopy.

## Supplementary Materials

Supplementary Materials Table S1: lentiviral plasmid backbones, lentiviral vectors, and miRNA mimics. Table S2: antibodies, their concentrations, and manufacturers in the present studies. Table S3: primers used for RT-qPCR. Supplementary Figure I: quantification of the expression of pmTOR, mTOR, and pS6K when primary cultured neurons were treated with NHIGDE, HIGDE, miR-199a-3p mimics, or miR-199a-3p shRNA.
