# Supplementary material for: Hypoxia-Induced Glioma-Derived Exosomal miRNA-199a-3p Promotes Ischemic Injury of Peritumoral Neurons by Inhibiting the mTOR Pathway
Source: Oxid Med Cell Longev. 2020 Oct 13;2020:5609637. doi: 10.1155/2020/5609637 (PMC7578720; doi:10.1155/2020/5609637)
Supplement: Supplementary Materials — Table S1: lentiviral plasmid backbones, lentiviral vectors, and miRNA mimics. Table S2: antibodies, their concentrations, and manufacturers in the present studies. Table S3: primers used for RT-qPCR. Supplementary Figure I: quantification of the expression of pmTOR, mTOR, and pS6K when primary cultured neurons were treated with NHIGDE, HIGDE, miR-199a-3p mimics, or miR-199a-3p shRNA. [file 5609637.f1.pdf]

Supporting information

Supporting Methods

1. Hematoxylin-eosin (HE) and hypoxia induced factor-1α (HIF-1α) staining

All tissue samples were fixed in 4% formalin, decalcified in ethylene diamine tetraacetic acid (EDTA) solution, and embedded in paraffin. They were cut into 4-μm thickness sections and heated in an oven for approximately 60 min. Then, the sections were deparaffinized with xylene and rehydrated followed by a graded series of ethanol. After heat-induced antigen retrieval and endogenous peroxidase blocking, the sections were incubated with primary antibodies overnight at 4 °C. Anti-HIF 1-α (ab16066, monoclonal, 1:400 dilution) were purchased from Abcam (Cambridge, UK). Subsequently, the slides were incubated with horseradish peroxidase-linked anti-mouse secondary antibody (ZB2305, 1:100 dilution, ZSGB-BIO, China) for 30 min at 37 °C. The universal 3,3'-diaminobenzidine detection kit (AR1026, Boster, China) was used as the final chromogen and the nucleus was counterstained by hematoxylin. Tissue samples were observed using a CX21 optical microscope (Olympus Optical Co., Ltd, Tokyo, Japan) after sealing.

2. Cell culture of HT22 and C6

HT22 and C6 glioma cells were cultured in DMEM supplemented with 10% charcoal-stripped FBS (HyClone, Logan, UT) and gentamicin (50 μg/ml) at 37°C in an atmosphere containing 5% CO_2 and 95% air. HT-22 and C6 glioma cultures were maintained at 50 and 100% confluency, respectively, in monolayers in plastic 75 cm^2 flasks.

3. Primary neuronal cultures

Rats were anesthetized with isoflurane and the E18 embryos were removed. The cortical region of the fetal brains was dissected in warm media and pooled together. The cortices were triturated and incubated in papain for 20 min at 37 °C, then centrifuged at 1500 rpm for 5 min at room temperature (RT). Cells were resuspended in MEM (minimal essential medium) (Gibco, Grand Island, NY, USA) containing 10% fetal horse serum (Hyclone, Logan, UT, USA), 2 mmol/l glutamine (Gibco), 25 mM glucose, and 1% penicillin/streptomycin (Gibco). Cells were plated onto poly-D-lysine-coated tissue culture plates at 7.5×10^5cells/ml. Media were completely changed after 24 h. One-half medium changes were performed at day 4. Cultures were incubated at 37 °C in a 5% CO_2 incubator and experiments were performed after days 9–11.

4. Oxygen-Glucose Deprivation (OGD) and Reperfusion

Aspirate the former medium in the 6-well plates 9hformer medium to simulate ischemia; the control should be disposed with BSS5.5 and incubated in 37°C, 5% CO_2. Then place the OGD-R groups in the Hypoxia Incubator Chamber, and 10-20 mL 2-time sterile H_2O to maintain the proper humidity within the chamber. Lock the clamp and open the inlet of the chamber and link the bacteria filter with the outlet tube of N2 Bottle. Next, open the switch and modulate the flow volume to 20L/min and sustain the nitrogen flow for 5min and then block the outlet and inlet of the chamber to maintain the low O_2 pressure within the chamber. Place the chamber into the 37°C incubator for 6 hrs. and control groups are incubated directly in the 37°C, 5%CO_2 incubator.

Fetch the OGD groups and aspirate the former BSS0. Well rinse twice with BSS5.5, and add the same volume of it as former BSS0 into wells. Incubate the OGD groups and control in the 37°C, 5%CO_2 incubator for 6 hrs.

5. 3-[4,5-dimethylthiazol-2-yl]-2,5-diphenyltetrazolium bromide (MTT) assay

For the purposes of the experiments at the end of the incubation time, cells were incubated for 4 h with 0.8 mg/ml of MTT, dissolved in serum free medium (MEM or DMEM for HepG2 and HTC cells respectively). Washing with PBS (1 ml) was followed by the addition of DMSO (1 ml), gentle shaking for 10 min so that complete dissolution was achieved. Aliquots (200 μl) of the resulting solutions were transferred in 96-well plates and absorbance was recorded at 560 nm using the microplate spectrophotometer system (Spectra max190-Molecular Devices). Results were analyzed with the Soft max pro software (version 2.2.1) and are presented as percentage of the control values. The materials were from Sigma Aldrich unless otherwise stated.

6. Transmission electron microscopy (TEM)

After the exosome samples were prepared as previously described, the purified exosomes were re-centrifuged using an Exosome Isolation Kit to collect exosome pellets. Briefly, each exosome pellet was placed in a droplet of 2.5% glutaraldehyde in PBS buffer and fixed overnight at 4°C. The exosome samples were rinsed 3 times in PBS for 10 min each and then fixed in 1% osmium tetroxide for 60 min at room temperature. Then, the samples were embedded in 10% gelatin, fixed in glutaraldehyde at 4°C and cut into small blocks (less than 1 mm3). The samples were dehydrated in increasing concentrations of alcohol, placed in propylene oxide and infiltrated with increasing concentrations of Quetol-812 epoxy resin mixed with propylene oxide for 3 h per step. Finally, the samples were embedded in pure, fresh Quetol-812 epoxy resin, which was allowed to polymerize at 35°C for 12 h, 45°C for 12 h, and 60°C for 24 h. Ultrathin sections were cut using a Leica UC6 ultra-microtome and stained with uranyl acetate for 10 min and lead citrate for 5 min at room temperature. The samples were then observed with a transmission electron microscope (NanoSight Ltd., Amesbury, UK) at a voltage of 110 kV.

7. Lentiviral vectors generation, titration and gene delivery

We used a 3 plasmids system for lentivirus packaging as detailed in our previous study, which include the lentiviral backbone containing miR-199a-3p shRNA and scramble shRNA genes, the packaging plasmid (p-delta) that provides all vector proteins driven by the trip CMV promoter, except the envelope protein, and the envelope-encoding plasmid (p-VSVG) that encodes the heterologous vesicular stomatitis virus envelope protein (VSVG). In brief, a mixture of 45 μg of transfer vectors, 30 μg of packaging plasmids and 15 μg of envelope-encoding plasmids were transiently transfected into 3 T175 flasks containing 1.5 × 107 HEK-293T cells using the calcium phosphate precipitation (CPP) method. Supernatants were collected 72 h post-transfection and viral particles were concentrated by ultracentrifugation. Viruses were resuspended in phosphate-buffered saline (PBS) and kept at −80 °C until use.

Virus titers ranged from 2×10^8 to 5×10^8 TU/ml and were diluted in PBS before gene transfer was conducted. The lentiviral vectors of GFP , S6K, HIF-1α, miR-199a-3p shRNA, scramble shRNA and diluted with PBS, were directly added in the medium of HT22, C6 cell cultures or primary neurons (10, 5 and 2 μl vectors were added into each well of 6, 24 and 96 well plates, respectively) with the multiplicity of infection at 1:5 (cells to virus units) immediately after medium exchange, and then incubated for 0–6 days. The same amount of PBS was also added to the medium as the experimental control (100 %).

8. Western blotting

Thirty micrograms of protein in each lane was subjected to SDS-PAGE using 4–15% Ready Gel (catalog #L050505A2; Bio-Rad, Hercules, CA) under 200 V for 45 min. Protein bands were transferred from the gel to polyvinylidinene fluoride (Millipore, Bedford, MA, USA) membranes under 100 V for 2hr. After blocked with Tris buffered saline containing 5%(w/v) bovine serum albumin and 0.1% Tween-20, the memberane was incubated with primary antibodies (supplementary table 1) incubated overnight at 4 °C followed by Alexa Fluor 488 donkey anti-rabbit or anti-mouse IgG secondary antibody (1:5 000, Invitrogen, Eugene, OR, USA) for 1hr in the dark room. Then membranes were scanned using Typhoon trio (GE Healthcare). The optical densities of all protein bands were analyzed using IMAGEQUANT 5.2 software (GE Healthcare). All samples were run on the same gel. The protein bands were rearranged solely to ease comparison in figures.

9. Reverse transcription-quantitative polymerase chain reaction (RT-qPCR)

Total RNA was extracted using TRIzol reagent (Invitrogen, Carlsbad, CA, USA), and cDNA was synthesized from 0.5 μg total RNA by reverse transcription using an ImProm-II reverse transcription kit (Promega, Madison, WI, USA) with random hexamer primers. Quantitative real-time PCR was performed using the SYBR Green QPCR system (Qiagen, Valencia, CA, USA) with specific primers. The PCR reactions were performed using an Applied Biosystems Prism 7000 sequence detection system. The level of target genes expression was normalized against the GAPDH gene. Data were analyzed with SDS 2.2.2 software using the $2^{-\Delta\Delta Ct}$ method with a relative quantification RQmin/RQmax confidence set at 95%.

Supplementary Figures Legends

Supplementary Figure I. Quantification of the expression of pmTOR, mTOR and pS6K when primary cultured neurons were treated with NHIGDE, HIGDE, miR-199a-3p mimics or miR-199a-3p shRNA. (A) When primary neurons were treated with NHIGDE + miRNA-199a-3p mimics, the expression of the mTOR pathway in primary neurons decreased compared with those in neurons treated with NHIGDE, similar to neurons treated with HIGDE after OGD/ reperfusion injury. *: $P<0.01$ vs. NHIGDE. (B) the levels of the mTOR pathway were higher in primary neurons cultured with HIGDE from C6 cells treated with miRNA-199a-3p shRNA than in neurons treated with HIGDE, similar to neurons treated with NHIGDE after OGD/ reperfusion injury. *: $P<0.01$ vs. NHIGDE. n=14-21/group in at least 3 independent experiments.

Supplementary Tables

Table S1. Lentiviral plasmid backbones, lentiviral vectors and miRNA mimics

Products	Manufacturer	Catalog#
miR-199a-3p shRNA	Addgene	#103319
Scramble shRNA	Addgene	#1864
Green fluorescent protein	Addgene	#17616
S6K	Addgene	#59446
HIF-1α	Addgene	#18949
miR-199a-3p mimics	Thermo Fisher Scientific Inc	MC11779
negative controls of miR-199a-3p	Thermo Fisher Scientific Inc	4464058

Table S2. Antibodies, their concentrations and manufacturers in the present studies

Antibodies	Source	Dilutions	Manufacturer	Catalog#
pmTOR (Ser2448)	Human	1:1 000	Cell signaling	2971
mTOR	Human	1:1 000	Cell signaling	2972
pS6K p70	Human	1:500	Cell signaling	9208
S6K p70	Human	1:500	Cell signaling	9202
CD81	Human	1:500	ThermoFisher	10616D
HIF-1α	Human	1:500	ThermoFisher	17-7528-82
β-actin	Human	1:3 000	sigma	A-5441

Table S3. Primers used for RT-qPCR

Gene	Forward Sequence	Reverse Sequence
miRNA-199a-3p	5′GCGGCGGACAGTAGTCTGCAC-3′	5′-ATCCAGTGCAGGGTCCGAGG-3′
mTOR	5′-TGATGGTGAGTGAAGAGCTGATTCGGGTAG-3′	5′-TTGGTGGACAGAGGGATGACAGCGTATCTC-3′
S6K p70	5′-ACTTCGGGTACTTGGTAAAGG-3′	5′-GATGTTCTCCGGCTTCA-3′

SPRINGER NATURE

Author Services

Editing Certificate

This document certifies that the manuscript

Hypoxia-Induced glioma-derived miRNA-199a-3p promotes ischemic injury of peritumoral neurons in glioma by inhibiting mTOR pathway

prepared by the authors

Jian-Lan Zhao, Bo Tan, Gong Chen, Xiao-Ming Che, Zhuo-Ying Du, Qiang Yuan, Jian Yu, Yi-Rui Sun, Xiao-Mu Li, Jin Hu, Rong Xie

was edited for proper English language, grammar, punctuation, spelling, and overall style by one or more of the highly qualified native English speaking editors at SNAS.

This certificate was issued on **March 16, 2020** and may be verified on the SNAS website using the verification code **D497-B0F7-7962-00B0-7632**.

Neither the research content nor the authors' intentions were altered in any way during the editing process. Documents receiving this certification should be English-ready for publication; however, the author has the ability to accept or reject our suggestions and changes. To verify the final SNAS edited version, please visit our verification page at secure.authorservices.springernature.com/certificate/verify.
If you have any questions or concerns about this edited document, please contact SNAS at support@as.springernature.com.

SNAS provides a range of editing, translation, and manuscript services for researchers and publishers around the world.
For more information about our company, services, and partner discounts, please visit authorservices.springernature.com.
